# On the Frequency of Asthenopia—Especially in America

**Published:** 1901-09

**Authors:** Lucien Howe

**Affiliations:** Buffalo, N. Y.


### On the Frequency of Asthenopia—Especially in America.

By LUCIEN HOWE, M. D., Buffalo, N. Y.

WE HAVE long since agreed that the term asthenopia shall be used to designate that familiar group of symptoms which includes discomfort or pain for near work with blurring of vision and perhaps some injection of the conjunctiva or lachrymation. But while we find this term convenient, it is also indefinite—especially so when we do not keep in mind the variety of asthenopia, whether accommodative, muscular, or central.

I. Remembering thus the general character of the term, the first fact to which attention is here directed is, that cases of asthenopia form one of the largest classes of any which we are called upon ordinarily to treat. Although this fact is so obvious, it is nevertheless difficult to demonstrate by figures, because the classification of diseases in institutions seldom or never includes the term asthenopia. It is possible, however, to approximate the number, for we know that nearly always asthenopia is associated with anomalies of refraction and of accommodation, and with muscular insufficiencies, and therefore the proportion of these cases gives with certain modifications, an indication as to the frequency of asthenopia. It is true that nearly all myopes and very many hypermetropes have no trace of asthenopia. But making allowance for these and also for those whose discomfort is trifling, there still remains so large a proportion of really annoying asthenopia as to make that group of cases in every enlightened country next in size to diseases of the conjunctiva and cornea. Indeed it is generally recognised that in private practice among the educated, the cases of asthenopia constitute by far the largest class.

II. Again, it is apparently the fact that the more annoying types of asthenopia, the muscular and central forms, occur more frequently in the United States than elsewhere.

As the term asthenopia is elastic, it would also be difficult to state the frequency of such cases in figures. I am not aware that any attempt has been made to do so, but it is an observation familiar to those who have had opportunities to compare the hospital or private practice in this country with that of England, France or Germany.

It is not intended to state that the average American is not as strong, in other respects, as his brother across the Atlantic. The contrary has been shown, as is well known, by Dr. P. C. Knapp and others. Dr. H. P. Bowditch has found from measurements of the American school boy that he is as good a physical type as the boy at Eton or Rugby. Dr. B. A. Gould in like manner found that our soldier was the physical equal of the European. Our athletes bring back at least their share of prizes from abroad and our yachtsmen have held the cup for over fifty years.

But, on the other hand, Javal has expressed the opinion to Roosa that asthenopia is more common in New York than in Paris, and this is also held by Bull, of Paris, whose extensive practice first on one side of the Atlantic and then on the other has afforded unusual opportunities for observation.

Important evidence on this point is furnished by Dr. George M. Beard in a monograph on Nervous Exhaustion and Neurasthenia, published so long ago as 1879. Most of the cases which he described are really types of asthenopia. In the first chapter he says, "there is a large family of functional nervous diseases which are increasingly frequent among the indoor classes of the civilised countries, and they are especially frequent in the northerly and easterly parts of the United States. The observations which compose the greater proportion of his monograph are carefully made and systematically arranged, and although some of the views advanced must necessarily be modified by the light of subsequent investigation, the conclusion which he reaches seems to be entirely warrantable.

He observes that the geographical distribution of this class of diseases is not everywhere the same; as they appear to be less common in Germany and Russia, Italy and Spain, more frequent in France and still more frequent in England. Above all, he considers neurasthenia to be an American disease and in that definition he includes, as before remarked, what we all recognise as forms of asthenopia.

The late Dr. Henry D. Noyes, of New York, presented as long ago as 1876, an analysis of 1079 cases of asthenopia which came under his observation—rather a large number even for an industrious practitioner.

Personally, as I compare the experience of twenty-seven years' practice here, and that obtained during ten visits among our confrères in England and on the continent, the impression becomes stronger, as before remarked, that such cases are decidedly more frequent in the United States. Moreover, it is natural that this should be so, for the following reasons:

(*a*) *Carelessness.*—Americans use their eyes more under unfavorable circumstances than is done elsewhere. Our newspapers and periodicals are many times more numerous and larger than in any other European country. The print is often poor, we read more when traveling or wherever we happen to be, in a few minutes of leisure, too frequently regardless of proper illumination or other requisites for the natural use of the eyes.

(*b*) *From causes affecting the nervous system.*—It is a saying with the Europeans that while he makes money to live, the American lives to make money, and however that may be, the hurry and mental strain of our American life makes greater demands upon the nervous system than does the more quiet routine of life elsewhere. This fact also is so well recognised as to require no comment.

(*c*) *From causes affecting the digestive tract.*—It is probable that this is of some importance. Americans not only eat more meat than do continental nations, but we do not take time to masticate it properly, and there may be an insufficient amount of fluids ingested as compared with that in wine or beer drinking countries. The fact that dyspepsia in various forms and appendicitis are so common in America, also indicate that there is some factor here which exerts an unfavorable influence upon the digestive tract and indirectly thus upon various organs.

III. Even if there should be a doubt that annoying forms of asthenopia do occur more frequently in the United States than in Europe, there is no doubt concerning another phase of this subject, which is, that a larger number of these asthenopic persons do apply for relief in the United States. This is especially apparent to German or English ophthalmologists who are so situated as to have a clièntele including many Americans.

Thus, one of our most honored and illustrious confrères, located in a German university town, which happens to be also a resort for Americans, said to one visitor, "your American eye-doctors frighten patients into glasses, and then the patients frighten themselves about them." In spite of an opinion from so distinguished an authority, it would seem that there are other causes which induce patients in America to seek relief sooner than patients do elsewhere. These are:

(*a*) The mental attitude of the average American in regard to glasses. He desires to see as distinctly as he can and prefers the inconvenience of glasses to any discomfort from asthenopia. This is true of American women also, young or old. This is even true, to as great an extent as is possible, of those other women of rather uncertain age, who naturally fear that the possession of a pair of glasses indicates that they are approaching middle-life. Especially is this objection lessened, when the optician assists in the self-deception by making the eye-glasses becoming or even ornamental.

(*b*) The average American practitioner is better supplied with appliances for detecting the causes and varieties of asthenopia than are his confrères in Europe. In addition to the all-important test case we find now almost invariably that he is equipped with an ophthalmometer, with a phorometer, together with other appliances for testing the static and dynamic conditions of the muscles, and occasionally has appliances for measurement of relative accommodation, which in Europe are to be seen only in a physiological laboratory.

When an American ophthalmologist visits his professional brethren in their offices in Germany, he is struck with the lack of appliances for properly testing this important class of cases.

(*c*) American ophthalmologists use these appliances to make more accurate corrections of ametropia and of muscular anomalies than is customary in Europe.

It may seem strange that the countries which have produced Helmholtz, Graefe, Donders and Javal should not also be those to carry into practice the important lessons taught at home. Apparently, however, that is not the case.

It required some time for American ophthalmologists as a class to be convinced that the correction of half or even a quarter of a diopter of astigmatism was of any importance. Gradually, however, the majority have reached that conclusion. We have learned that attention to the details of ametropia and of heterophoria, apparently insignificant, make all the difference, in some few instances, at least, between persistent headaches and perfect comfort.

(*d*) The skill of the optician assists to no small degree in the treatment of these cases of asthenopia. In nearly all of our cities of any considerable size we now find one or more opticians who themselves grind spheres and cylinders accurately and promptly, setting the glasses in frames which are neat and comfortable.

They have long ago learned to make bifocal lenses, so that certain hypermetropes or myopes who have passed middle-life find such lenses one of the essentials of comfortable existence. And yet, even this simple appliance is exceptional, in a country where the theory of optics and the skill of the workman otherwise is as far advanced as in Germany. During the last year the writer of this, who wears bifocal lenses, attended the meeting of the German Ophthalmological Society in Heidelberg. Such glasses were so unusual as to attract attention and several of the members did not hesitate to express their curiosity by inquiries concerning them. We must conclude, therefore, that cases of asthenopia form not only one of the largest groups which we are called upon to treat, but that the proportion of severer cases is somewhat larger in America than in other countries, this being due to the injurious effects of our national habits upon the eyes themselves, upon the nervous and digestive system.

Also, it seems quite certain that a larger proportion of all the cases of asthenopia apply for relief in the United States than do so elsewhere, the reasons of this being the comparatively slight aversion of patients to the use of glasses, to the fact that the average American ophthalmologist is better equipped than his foreign confrères for detecting errors of refraction and of muscular balance, that he takes pains to correct even small variations from the normal condition, and that the American optician has much more complete appliances for grinding glasses accurately, and fitting them satisfactorily, than has the average optician of any other country.

183 Delaware Avenue.

